# Practical Observations on Diseases of Women

**Published:** 1839

**Authors:** 


### PRACTICAL OBSERVATIONS ON DISEASES OF WOMEN. By *William Jones*, M.R.C.S. Surgeon to the Free Dispensary and Infirmary for Women, &c. and Lecturer on the Principles and Practice of Midwifery, and the Diseases of Women and Children. Illustrated with Cases and Explanatory Plates. 8vo. London, H. Balliere.

THE object of this work may be gathered almost as perfectly from its preface and table of contents, as from a diligent perusal of it. It is the urgent recommendation of the use of the speculum in uterine affections. Nor is that object circuitously sought or insufficiently estimated. Mr. Jones puts the highest value on it. Nothing would gratify him more than *speculations* on, we should say *in*, the cases of our wives, our sisters, and our daughters. "He has," so he informs us, "too frequently had to deplore the ignorance of those indications of the real nature of uterine diseases, attainable only by physical examination in its most extended sense, when he has seen an unfortunate patient insusceptible of relief from human skill, gradually sink before his view, while yet 'in the full pride of youth and bloom of womanhood.' Too frequently, also, has he had to encounter the many difficulties connected with the employment of the means he advocates, to be unacquainted with the prejudices of the public respecting it; but, in surmounting them, he has perceived the force of the maxim of Cicero,—'Whatever we utter with truth, although it be praised by none, is by nature commendable.'

Another motive by which the writer has been induced to offer these observations to the consideration of his professional brethren, is the desire of bringing together a mass of facts in proof of the necessity and utility of this mode of examination, and of having an opportunity of requesting their assistance in the formation of an establishment for a fair trial of its merits in every point of view." iii.

With every disposition to approve of exact and physical means of diagnosis, we must say that we think Mr. Jones either a very unhappy or a very fortunate man, in having *frequently* seen ladies "in the full pride of youth and bloom of womanhood" sink before his view, because the speculum had not been used on them. It has not been our lot to see many such cases, and we experience some difficulty in conceiving what they could have been. Mr. Jones's experience must evidently have been considerable.

Mr. Jones's great aim is to foster an institution for the employment of the speculum. A gentle periphrasis oils the instrument—the institution being established for "the physical examination of the fair." Let Mr. Jones champion it and them.

"In requesting the assistance of his professional brethren in the accomplishment of this important object, the author solicits the exertion of their interest, rather than their pecuniary assistance, well aware how innumerable are the calls that are daily made on their benevolence; and knowing also that, in the promotion of any charitable design, they possess a moral power not exceeded by that of any other body of men. Could they, as a body, but clearly perceive its utility and necessity, he feels convinced that a few months only would elapse ere such an Institution would do honor to our Metropolis; and, in truth, is not every professional man personally interested in the formation of such an Institution? Could such an Institution exist for any length of time, without conferring benefit on him, his family, and the public at large? Would he not daily have an opportunity of obtaining information which could never be acquired in the ordinary routine of private practice? Would not his wife, his daughters, his patients, and his friends, derive the benefit of that information? Would not many persons be rendered comparatively happy, or at least free from disease, who are now its unfortunate victims? Surely these questions cannot but be answered in the affirmative.

But is the public less interested in the accomplishment of such design? No; every man, no matter what may be his station in life, who has a mother, a sister, a wife, a daughter, or any other female relative,—every woman whose bosom glows with benevolence, or whose heart can feel for the sufferings of another,—is bound, by all that renders life desirable, to assist in the promotion of the charitable design." v.

It must be clear that results of a very high description would flow from a speculum hospital. The Metropolis would acquire an accession of honor (the very pox hospital would barely do it as much)—domestic happiness would receive a sensible increase—and every man who has a female relative, (a large and influential class) as well as every benevolent woman, is bound, by all that renders life desirable, to lend a helping hand. This adjuration deserves to be successful, and we should not be surprised if a strong enthusiasm is excited in the public mind. To give effect to Mr. Jones's call upon the "masses," it might not be a bad plan to suggest its incorporation with the "Charter." Perhaps "Doctor Fletcher" would move this in the

National Convention. It would not be unadvisable for every practitioner interested in the speculum cause, to enlist all his patients, and particularly his female patients, in its favour. He might carry one in his pocket, and explain in easy and familiar terms its uses, application, and advantages over the "touch." On married ladies, widows, or spinsters beyond a certain age, the practitioner might offer to manipulate. Nothing would carry conviction like personal experience, and for the sake of the sex, what man would not do as much as this?

Unreflecting persons may think the speculum a very beastly instrument. But what says Mr. Jones?

"Far, far from him be the wish to violate, or to encourage the violation of those habits of modest and delicate sensibility, which throw a halo round the sex, and give grace and dignity to the most imperfect form. May it ever be his earnest wish, as it has hitherto been his constant practice, to uphold the dignity and protect the delicacy of the sex, whilst reluctantly compelled to resort to those means, which are absolutely necessary to the mitigation of its sufferings, in many of their severest forms." vi.

At first sight it would appear a difficult matter to uphold a lady's dignity, at the instant when, on her back upon a table, with her thighs wide open, and her knees up to her chin, a gentleman of sad demeanour is curiously peering up her vagina with the assistance of a wax, or a tallow candle. But the conscientious surgeon and the enthusiastic patient may find a moral dignity in their mutual relations which escapes ordinary observation.

Mr. Jones is impressed with the vastness of his plan. Let him but get the hospital upon its legs, (perhaps the metaphor should change positions and lie upon its back) and he will die with pleasure. Such devotion requires to be signalized; it is not common in these days.

"In conclusion, if any thing the writer has advanced, have the effect of inducing a single practitioner to resort more frequently to physical investigation, in forming diagnoses of diseases, especially of women;—should it ever be his lot to see the sapling Institution, whose cause he has advocated, become the perfect tree, affording shade and shelter to all that come within its reach;—then will his object have been attained,—then will he have no reason to regret his labor, but will 'lay him down in peace,' happy in the assurance of having contributed in some small degree to the alleviation of those sufferings, which fall with peculiar severity upon the more amiable portion of humanity." viii.

If the ladies are not sensible of the chivalry of Mr. Jones, they deserve never to have a speculum employed upon them. He offers (can he do more?) to die in their behalf. Only let him put them in a fair train for examination—only let him make "physical exploration" fashionable—only let him once see specula popular—and then, like the prophet, he will lay him down in peace. Ladies of England, can you refuse to pull up your petticoats at such solicitation?

The Work consists of Two Parts. The first is on the Necessity of Physical Examination, and on the use and application of the Speculum. This part contains five chapters. They are severally intituled :—

Of Physical Examination in cases of disease generally—Of Physical Examination as applied to Diseases of the Uterus in particular, and of the various modes of performing the Taxis—Of the Physical signs recognizable by the Taxis in the healthy uterus;—in the unimpregnated state,—during

Gestation,—and after Parturition—Of the Speculum—its use and application; and of the Physical signs appreciable thereby—Of the objections which have been advanced against the use of the Speculum.

The second part is made up of fifty-two cases, which occupy more than one half of the work.

Passing over the three first chapters, which contain nothing authoritative enough to arrest us, we pause at the fourth, which treats of the Speculum, and of the physical signs appreciable by it. We copy our author's observations on the instrument that he prefers, and on the mode of using it.

"The instruments I have generally found sufficient for all practical purposes, are Recamier's metal tube, or a simple glass tube of the same dimensions,—Ricord's two-bladed Speculum—and, very rarely, Weiss's Speculum with four blades; and of these, the one I employ most generally, is the two-bladed instrument of Guillon or Ricord. Recamier's speculum is principally of value in cases wherein it is desirable to apply caustics, leeches, and other applications to the cervix uteri, and we wish at the same time to prevent them from coming into contact with the vagina; Ricord's two-bladed instrument, or Weiss's instrument with four blades, is advantageous in the examination of women who have borne children, and in whom the relaxed vagina, protruding through the three-branched instrument, prevents the free inspection of the uterus. The dilator is most applicable to younger females, or to cases wherein it is desirable that the condition of the vagina should be known, as in Vaginitis, &c. Whatever may be the form of instrument employed, the female should be made to assume the recumbent position, with the pelvis somewhat elevated,—the thighs and legs bent and separated, so as fully to disclose the genital organs. When (having previously immersed the instrument in warm water, so as to prevent the sensation of cold, which usually follows the application of metal to the surface or interior of the body, and having covered it with spermaceti ointment, or cold cream, so as to facilitate its passage within the vagina), the operator proceeds to its introduction:—

In the first place he separates the labia externa and nymphæ, with the thumb and second finger of the left hand, so as to disclose the orifice of the vagina, and retains them separated, until the index finger (previously oiled) be introduced within the vagina;—the index finger having reached the cervix uteri, points out the locality of the uterus, and serves as a guide for the speculum, while it makes pressure on the lower part of the vagina, and dilates its cavity; meanwhile, the speculum, held in the other hand of the operator, is gradually conducted on the upper surface of the finger, along the vagina, till it reaches the cervix uteri,—the finger being gradually withdrawn as the speculum advances. The depression of the fourchette and perinæum by the finger *in vagina*, not only dilates the cavity of the vagina, but prevents contusions of the meatus urinarius, which might otherwise result, from its being compressed between the instrument and arch of the pubis. The proper position of the patient may be obtained by placing the patient across a bed, bringing the pelvis close to its side, and supporting it and the head, by means of pillows, but it cannot be so readily attained as by means of a table which I have contrived for the purpose, and of which the subjoined is a rough outline. It consists of a frame, supported on four legs, to which a cushioned top is attached by hinges at one extremity; by means of these hinges and a rack placed beneath it, the top of the table may be made into an inclined plane, so as to give to the pelvis the requisite elevation; by means of the cushion for the head, those inconveniences may be obviated, which might result from retaining the head in the dependant position. To prevent the legs of the patient getting in the way of the operator, two iron rods with semicircular cushions are attached to the table, over which the legs are placed;—these rods are moveable, and are capable of giving different lengths, to correspond with the varied extent of the *femora* of different females. The operator, seated in front of the table, introduces the instrument in the manner described, and if the operation be performed in a light room, and the woman be placed facing the light, the cervix uteri is fully exposed, and its condition easily appreciated. In other cases it is necessary to employ the light of a taper. The complicated instruments, with mirrors, recommended by Segalas and others, or even the employment of a mirror of any kind, is not only unnecessary, but may, by the refraction it occasions, induce incorrect ideas." 71.

We add the following brief statement of the conditions of the cervix uteri, under the circumstances pointed out:—

"If the unimpregnated uterus be examined by the speculum, it cervix is found to be of a pale rose red colour, in young females; of a brighter hue in middle-aged women; and of a faded greyish pink colour in women advanced in years. It becomes swollen, and is found to be of a darker red, at, and immediately antecedent to each menstrual period. Externally it is always moistened with a coat of thin mucus; its orifice, on the contrary, frequently contains a small quantity of viscid fluid, secreted by the vesiculous glands, or lacunæ (*glandulæ nabothi*), which are situate within its cavity. The membrane lining the cervix uteri in health, is of the same colour as that covering the vaginal surface. During gestation, the latter months especially, the membrane covering the cervix uteri, and lining it and the upper part of the vagina, is of a purplish hue; probably resulting from the impediment offered to the free return of the blood from the extremities, by the pressure of the gravid uterus; or rather from the congestion necessary to the supply of materials to the fœtus *in utero*." 72.

The fifth chapter is on the objections which have been advanced against the use of the speculum.

It may well be believed that Mr. Jones sees little force in the objections to the speculum, much in the arguments for its employment.

Vision he argues to be an important sense. The eye, he says, is the organ which affords the clearest and most important indications, in every disease which can come within its scope in the living subject;—the lynx eye it is which constitutes the great accomplishment of the physician; and without an eye he would rarely be consulted even by the public, much less by his professional brethren. All this may be safely granted, and yet it does not go far towards the settling of the matter. Nobody would shut his eyes when he could unobjectionably open them. But it is contended that the indecency of the speculum is not counterbalanced by equivalent advantages.

Mr. Jones, however, is not of this way of thinking. He is an unflinching advocate of the employment of the instrument, and not more unflinching than successful. So, at least, it would appear from the following passage.

"Women, it is asserted, will not submit to the employment of the speculum. To this assertion, however, I must beg to offer my unqualified denial; because for the last four years I have myself been engaged in the daily (I might almost say hourly) use of the speculum, as well in private as in dispensary practice, alike in the humbler, the middling, and the higher classes of society; and I have rarely met with the objections and prejudices which some practitioners assert to exist against its use. Indeed, I have rarely found women those senseless beings which some individuals would induce us to believe them; and I have yet to discover one, even one, who would prefer retaining a loathsome and perhaps fatal disease, to submitting to any examination for its removal, provided the nature and object of the examination were properly explained to her, and she were, by such explanation, convinced of its necessity, and of the total impossibility of adopting any scientific treatment, without its employment." 91.

The secret of his good fortune is discovered by himself. He does not rudely assault delicacy, he gently overpersuades it. In this he appears to be a master. Few women, we should imagine, could resist his insinuating advances. The ladies, we are confident, vote him unanimously " a dear man." Listen to "the artful dodger."

" Fully convinced of the total impossibility of forming correct diagnoses in diseases of the uterus, or its appendages, without the assistance of physical examination, I have long since felt the equal impossibility of commencing a scientific treatment without its employment; and whenever I have reluctantly yielded to the wishes of individuals, or to the common usages of the profession, by *commencing* a treatment founded on the obvious existence of certain derangements of function, without a knowledge of the alteration of structure on which they were dependant (as I have frequently been compelled to do,) it has been solely for the purpose of gaining the confidence of the patient, and of seizing at some subsequent period (provided the symptoms did not yield to the palliative treatment I might chance to employ,) a proper opportunity to explain to her that the symptoms observed might result from any one of a great number of different diseases, of which the particular affection which gave rise to them in herself, could only be determined by physical examination, and to solicit her submission to its employment; telling her, at the same time, that without a knowledge of the nature of her disease, we might continue our hazardous treatment for months or years in succession,—might exhaust the whole range of the materia medica in our fruitless efforts to relieve her, and find at last, too late, that instead of mitigating her symptoms, we had only been aggravating her disease, and converting a simple and easily treated affection into a formidable, appalling, and perhaps fatal malady." 97.

What petticoat could be closed to such solicitation? We might as well try to find " a cat averse to fish," as a female obdurate to such a prayer. The representations contained, may be, perhaps, un peu trop fort. But what of that? Is eloquence to be chained to the slow and heavy wheel of mere demonstrable fact? It would lose its very name and being. The pleader's art may be fairly essayed in the great cause of the speculum.

It will be found that Mr. Jones's success has emboldened him to apply the instrument pretty liberally. Maid, wife, and widow, are fish for his net. He would apparently, if he could, start with a speculum in every case in which " the parts " have a flaw. From amenorrhœa upwards, all affections require, or justify the use of the instrument—all, excepting such cases of morbid growth, as present a palpable and sensible alteration. This is a pretty liberal code. Our young ladies have a treat in store if it prevails. The Ovidian recipe for fractured virginities must needs grow rather fashionable. This professional mode of taking a maidenhead will not, we fear, be much in vogue with the male sex. As Mr. Jones observes, such an objection too frequently arises from the said males' sensuality. We quote, in order that we may not unintentionally misrepresent our author.

" Another objection which has been raised against the speculum is, that it may be used unnecessarily, and may not afford a single symptom that could not have been discovered by other means; but this objection is untenable, for negative evidence is frequently as valuable as positive; if, for example, a patient had complained of profuse discharge from the vagina, and on examination with the speculum, the vagina should exhibit no change of structure to account for it, we should thence infer that it did not proceed from disease of the vagina itself, but from the suffering communicated to it by some neighbouring or distant organ, and we should then have to determine what organ it were whose derangement had given rise to it; the speculum is not *therefore* unnecessary, but it is unnecessary, *when it cannot be made use of;* when, for example, a simple disease has been allowed to proceed insidiously, unnoticed or disregarded, and has filled up the whole of the vagina with a morbid growth,—or when in the advanced stage of cancerous affections, the vagina is contracted by the deposition of the morbid material, and the finger passed over it instantly recognizes that peculiar sensation characteristic of cancer or scirrhous disorganization, which must be felt to be appreciated, but which having been once felt can never be forgotten. In every other case it is necessary; for, although it be true that we may recognize the presence of a tumour by the touch, we cannot determine the nature of that tumour without ocular examination, and should confound (as frequently has been done) fungus with polypus, scirrhous with simple induration, &c." 99.

Mr. Jones warms as he proceeds. In summing up, he announces that the practitioner who does not use the speculum incurs "an awful responsibility." Those who are tainted with professional levity may fail, perhaps, to perceive this, and may remain very unconcerned at their serious situation. Mr. Jones evidently feels it no joke, and he concludes in a strain, borrowed as he confesses from Dr. Blundell, which is of a very animated and impressive nature.

"'The more the discussion, the more objection and defence it has to undergo, the better. If it be grounded in error let it perish; if in just principles, it must survive. From the most violent conflicts of opinion truth has nothing to fear; though long to us, to her a thousand years are but as one day—a point—a nothing in the eternity of her duration. Oppressed amongst us, beneath the chaos of human follies and errors, she must, she will emerge unhurt at last—unchangeable as her Author. By the mere force of durability she must ultimately stand alone, solitary amid the wreck of those perishable materials by which for a time she is overwhelmed.

> The stars may fade away—the sun himself
> Grow dim with age, and nature sink in years ;
> But she—

the living spirit of philosophy, immutable, immortal, infinite, eternal TRUTH, she, the parent of all knowledge, fountain of light,—

> ———will flourish in eternal youth
> Unhurt, amid the war of elements,
> And wreck of worlds.' "

Of course it is not meant that the speculum will literally flourish unhurt, amid the wreck of worlds. This only expresses Mr. Jones's anticipations in a figurative manner. It is a peculiar feature of the speculum, that it breeds a highly poetical strain in all who advocate its introduction. They assume the lover's ardour, and seem almost to have "eaten of the insane root." Our readers may remember the paroxysm of fervor into which Dr. Balbirnie worked himself. Here is Mr. Jones, in a mood almost as poetical. He does not, indeed, like the Doctor, proclaim himself, the "Apostle" of the Speculum, but he announces how willingly he would die for it. Is there any other instrument of which we could say so much. Who offers to die for a bougie, to be the apostle of the "cutting plyers?" The charming sex to which the speculum is devoted, has shed its enthusiasm on the votaries of the speculum.

Dropping the tone of badinage, we may briefly express our opinion of the instrument. We do think it over-rated by gentlemen like Mr. Jones, while it is not in general sufficiently resorted to in practice. But we cannot conceal from ourselves or from the patient, the indecent and disgusting nature of the process of employing it, nor will we conceal our impression that the cases which require it, are much, very much less frequent than writers like Mr. Jones or Dr. Balbirnie, represent. We bring the subject before our readers, in order that the discussion to which it gives birth may lead to a more stable, and an experimental appreciation of the real service the speculum is likely to perform. That service, we are sure, would not be inconsiderable, were there not the drawback of the extreme indecency which sticks to the employment of the instrument. That will, we apprehend, form a bar to its extensive use in this country, in the middle and upper classes of society.

We hardly know whether to regret this circumstance. If science loses by such delicacy, morality and the interests of society gain, and, perhaps, after all, the balance is even. We are quite sure that the advocates for the speculum over-rate its utility and exaggerate its necessity. The other means of diagnosis though avowedly, in some instances, imperfect, are in the great majority, adequate to the occasion.

We have only to add that we have no intention of giving pain to Mr. Jones, by any tone of ridicule which may have tinged our observations. The subject, or rather the mode of handling it, invites it, and enthusiasm on an author's part, carried past a certain limit, requires the pruning-knife of gentle satire. The merit of enthusiasm we freely award to Mr. Jones, and probably his next essay will be sobered by discretion and experience.

